# An Investigation of the Effect of Novel Mono/Bi-Layered PVD-Coated WC Tools on the Machinability of Ti-5Al-5V-5Mo-3Cr

**DOI:** 10.3390/ma17153743

**Published:** 2024-07-28

**Authors:** Hasan S. Syed, Jose M. DePaiva, Victor Saciotto, Stephen C. Veldhuis

**Affiliations:** McMaster Manufacturing Research Institute (MMRI), Department of Mechanical Engineering, McMaster University, 230 Longwood Rd S, Hamilton, ON L8P 0A6, Canada; paivajj@mcmaster.ca (J.M.D.); saciottv@mcmaster.ca (V.S.); veldhu@mcmaster.ca (S.C.V.)

**Keywords:** machinability, Ti-5553, PVD coatings, tribology, cutting tool, AlCrN

## Abstract

The Ti-5Al-5V-5Mo-3Cr (Ti-5553) alloy is a relatively novel difficult-to-cut material with limited machinability and tool life analysis available in the literature, and hence requires further investigation. This study focuses on the machining and tribological performance of Ti-5553 under high-speed finish turning (150 m/min, 175 m/min, and 200 m/min) via novel mono/bi-layered PVD-coated WC tools. A base AlTiN coating is used as the reference monolayer coating, with AlCrN, diamond-like ta-C, and TiAlSiN coatings each deposited on top of a base AlTiN coating, totaling four separate coated tools (one monolayer and three bi-layer). Tool life, cutting forces, workpiece surface quality, and tribological chip analysis are among the subjects of investigation in this study. Overall, the AlTiN/AlCrN coated tool outperformed all the other combinations: an improvement of ~19% in terms of tool life in reference to the base AlTiN coating when averaging across the three speeds; lowest surface roughness values: ~0.30, 0.33, and 0.64 µm; as well as the lowest chip back surface roughness values: ~0.80, 0.68, and 0.81 µm at 150, 175, and 200 m/min, respectively. These results indicate that the AlTiN/AlCrN coating is an excellent candidate for industrial applications involving high-speed machining of Ti-5553.

## 1. Introduction

The VSPO-AVISM Corporation developed a metastable (up to ~850 °C) body-centered cubic (BCC) near-beta Ti alloy (commercially branded as titanium–molybdenum alloy or “TMA”) called Titanium-5Al-5V-5Mo-3Cr (Ti-5553) that has excellent properties such as high strength, high toughness, exceptional corrosion resistance, elevated temperature stability, and is reported to be effectively lighter than steel- or nickel-based alloys, providing an excellent strength-to-weight ratio [1,2]. Ti-5553 at present is commonly utilized in the aerospace industry, particularly in landing gears (Boeing 787 Dreamliner and Airbus 350), and to a certain extent in biomedical applications, 3D printed implants being an example [3,4]. The superior mechanical properties achieved through the formation of the metamorphosed β-phase and higher concentration of certain elements over Ti-64 facilitates its serviceability in heavy duty applications; however, these very properties hinder its machinability and in turn result in worsened tool life and workpiece surface quality, a decrement of ~25% in terms of metal removal rates, and ~15% lower tool life [5,6,7,8].

Ti-5553 belongs to the β-alloy group, unlike the most commonly used Ti-64 alloy, which is categorized as having the α + β microstructure. It has a metastable β-structure and comes under the class of near-beta alloys due to beta stabilizers (V, Mo, Cr, and Fe), shown in Table 1, which lower the temperature required to stabilize the beta phase and contribute to the superior mechanical properties (~15–30% higher tensile and yield strengths) that Ti-5553 has over Ti-64 (Table 2) [9,10]. Compared to the 80% α-phase concentration in Ti-64, a proportion of only 20% exists in Ti-5553 in the form of α + β Widmanstätten, or bimodal/globular residual α microstructure [10]. In addition, the thermomechanical properties of any given alloy are not only defined by the microstructural phases, but also the chemical composition of the integrated elements. Table 1 and 2 show the elemental composition and mechanical properties, respectively, of the Ti-5553 alloy used in this study (Grandis Titanium), and a typical Ti-64 alloy. The elements involved in the alloying of a near β-titanium alloy are generally categorized as β-stabilizers, α-stabilizers, and neutral [11,12].

Machining Ti-5553 at any cutting speed yields chips that have narrow adiabatic shear bands, unlike Ti-64, which forms partially segmented chips without any adiabatic shear bands (transitional chips) at low cutting speeds and segmented with adiabatic shear bands (also known as catastrophic thermoplastic shear [18]) at high cutting speeds. Moreover, these adiabatic shear bands can cause oscillations in force components resulting in rapid tool wear [10]. The size and shape of the chips are highly dependent on controllable machining factors, and analyzing the chips can aid in understanding the dynamics that contribute to the formation of chips [19]. The shear bands and back surface roughness are some of the factors that can be analyzed to give us details in relation to their effect on chips. Chips that are curly, thin, and have a low undersurface roughness, for instance, would be indicative of low friction at the tool–chip interface and improved chip flow [20].

Cutting forces and their components are amongst the major criteria for evaluating the machinability performance to aid with optimizing and improving machining of Ti-5553. The highest mechanical stresses due to cutting forces are found in the region of the cutting tool–chip interface due to the resistance to deformation (brittleness) of titanium alloys at high temperatures [12]. Increasing the feed rate, depth of cut, and cutting speeds all lead to an increase in the cutting forces. However, at low depths of cut, varying the cutting speed has little influence on the cutting forces, whereas the opposite is true at high depths of cut. Increased variability in cutting forces with changing cutting speeds at high depths of cut is indicative of tool wear rate influencing the requirements for the cutting forces to form chips [21]. Moreover, cutting forces can be influenced by cyclic loading resulting in chatter (common in finishing operations) and eventually wear, and is more prominent in β-alloys [18]. Additionally, Ti-alloys such as Ti-5553 experience deflection almost two times that in carbon and stainless steels, which can result in the early onset of flank wear due to larger machined surface spring-back [12]. For this study, a low depth of cut was chosen to minimize the impact of cutting force variation with increasing speed in order to reduce the number of variables.

The highest temperatures in the machining of Ti-alloys are observed in the primary and secondary shear zones (tool–chip interface and tip). It has been reported by Kaynak et al. [21] that for Ti-5553 alloys, the cutting temperatures increase with an increase in cutting speed. As for the depth of cut, it causes the temperature to increase with its increase, with the highest influence at cutting speed above 120 m/min (for tungsten carbide tools (WC)). Moreover, the tool’s hardness can decrease due to thermal softening at high temperatures, and stresses at the cutting edge, which can cause plastic deformation and extreme wear [18]. The effect of feed rate is marginal as compared to the depth of cut and cutting speed.

The surface integrity of Ti-5553 is primarily why it is used in applications requiring excellent reliability; hence, it is crucial to ensure that the surface finish is monitored and kept within an optimal range. Kaynak et al. [21] mention that for Ti-5553 alloys, increasing the depth of cut results in an increase in surface roughness, whereas increasing the cutting speed decreases the surface roughness, owing to high tool wear, and a worn cutting tool nose giving a smoother surface finish, respectively. Although with increasing cutting speed the surface roughness of the workpiece decreases, its dimensional accuracy deteriorates due to the worn cutting tool nose.

The type and material of cutting tool utilized are highly dependent on the workpiece being machined and the limitations imposed by the equipment, amongst other factors. To resist sudden failure, the cutting tool material should have high resistance to abrasion, fracture toughness, and resistance to plastic deformation. The chipping resistance as well as the feed rate and depth of cut permitted are contingent on the cutting tool’s resistance to fatigue and fracture. As for the maximum allowable cutting speed, the tool material is required to have high thermal conductivity, chemical inertness, and high hardness (i.e., penetration resistance) [22]. Coatings are applied as a means to enhance the properties of the surface layer of the tool material. Depending on the application, they can be applied to reduce the friction coefficient, improve the thermomechanical properties, or resist corrosion and oxidation. The primary focus of this discussion is on thin coatings (0.1–10 μm) deposited via physical vapor deposition (PVD).

The available literature primarily pertains to the challenges faced with the use of uncoated WC tools during the turning machining of Ti-5553. Limited research is available on the use of coated tools, or a comparative analysis of PVD-coated WC tools for cutting Ti-5553, as is evident from Table 3. A comprehensive study comparing coatings with different chemical compositions and architectures in terms of their performance for high-speed machining of Ti-5553 has not been reported. Hence, this study delves into performing a comparative analysis of single and bi-layered PVD-coated WC tools in the finish turning of Ti-5553 in relation to tool life, surface quality, and tribological conditions. Highlighting the effect of the coatings’ micromechanical properties, such as hardness, H/E, H3/E2, plasticity index (PI), and the importance of reducing the adhesion levels during the machining of this type of material.

## 2. Materials and Methods

### 2.1. Experimental Setup and Machining Studies

**Figure 1:**
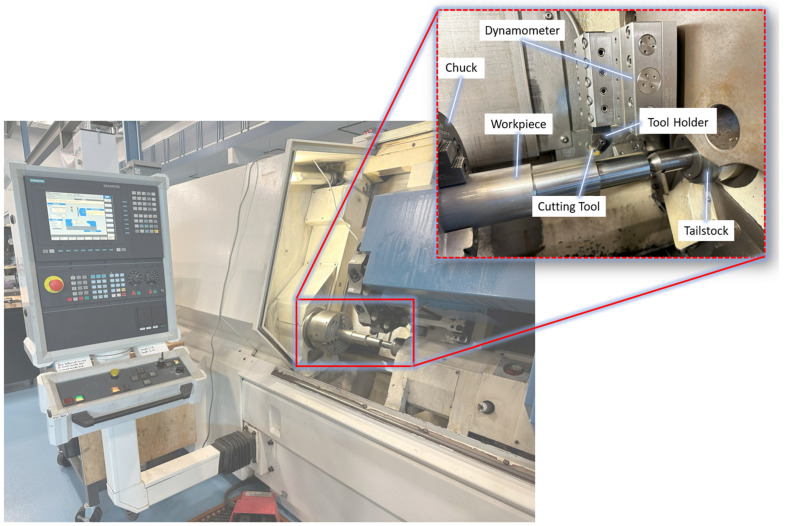
Experimental machining setup for machining of Ti-5553.

Sets of single and bi-layered coated Kennametal CNGG120408FS K313 cutting inserts were used in the finish turning of Ti-5553 alloy supplied by Grandis Titanium, under flood lubrication with a coolant concentration of ~9%. The Boehringer VDF 180 CM CNC (FFG Europe & Americas, Eislingen, Germany) turning lathe was the machine employed for this study (Figure 1), with the cutting parameters and tools used provided in Table 4. To measure cutting forces, a piezoelectric Kistler Type 9129AA 3-component dynamometer was mounted to a Kenloc^TM^ MCLNL164D NJ9 5° tool holder. The analog data was fed into a National Instruments (NI) Type 9215 data acquisition card, converted to digital, and then acquired by the National Instruments (NI) cDAQ-9172 DAQ system. The data were processed via a National Instruments (NI) LabView 2014 data analysis system collected at a sampling rate of 10 kHz.

### 2.2. Coating Deposition and Cutting Tools

A monolayered commercial AlTiN-based (BALIQ^®^ ALTINOS, Lake Orion, MI, USA) coating was used as the reference, with TiAlSiN (Kyocera HDT, Cuyhoga Falls, OH, USA), AlCrN (Ionbond Crosscut^TM^ Plus, Duncan, SC, USA), and DLC ta-C (Ionbond Tetrabond^TM^ Plus, Duncan, SC, USA) coatings deposited on top of the base AlTiN layer, for machining and characterization tests. Kennametal CNGG120408 K313 inserts were used for the cutting tests whereas polished WIDIA SNUN120408 WC coupons were utilized for the micromechanical and characterization tests to assess coating performance. The base monolayer coating designed for turning was deposited using s3p (scalable pulsed power plasma) HIPIMS technology, which incorporates both arc and sputtering technologies, resulting in excellent surface quality with minimal macroparticles. The AlCrN layer is an extremely versatile coating suitable for usage under both dry and wet cutting conditions at temperatures up to 1050 °C. The ta-C coating is optimal for the machining of non-ferrous metals such as titanium alloys and can be operated at temperatures in proximity of 500 °C. Both, having been deposited by *Ionbond*, employed PVD Advanced Arc Technology. The TiAlSiN coating was engineered to provide high performance solutions for titanium and super alloys at operating temperatures exceeding 1000 °C.

The WC coupons were hot-mounted in a black phenolic powder supplied by the MetLab Corporation (Niagara Falls, NY, USA) in a SimpliMET^TM^ XPS1 Compression Mounting System (Lake Bluff, IL, USA). The mounting procedure was as follows: The heat and cool times were set to 10 and 5 min, with the maximum temperatures and pressures being 170 °C and 3.65 psi, respectively. Thereafter, a Struers Tegramin-25 semi-automatic machine (Copenhagen, Denmark) was used to polish the mounted sample in four steps: Step (1) disc speed: 300 rpm; force: 30 N; holder speed: 180 rpm; lubricant type: water; and duration: 6 min. Steps (2–4) disc speed: 150 rpm; force: 30 N; holder speed: 150 rpm; duration: 5 min; lubricant type: DP-Lubricant Blue with 9, 3, and 1 µm diamond suspensions; in series for steps 2, 3, and 4, correspondingly. After attaining a smooth reflective surface, the samples were coated.

### 2.3. Tool Wear Measurements

The VHX-5000 Keyence optical microscope (KEYENCE Corporation of America, Elmwood Park, NJ, USA) was used to inspect flank wear progression in increments of 30 m linear passes, with failure marked at 300 µm wear land in accordance with the ISO 3685:1993 standard [36]. The focus variation technology of the Alicona Infinite Focus G5 3D surface measurement system (Alicona Manufacturing Inc., Bartlett, IL, USA) was used to analyze cutting edge wear and create volumetric datasets using the EdgeMaster and MeasureSuite modules. In order to create and compare the 3D volumetric transformation of the tools before and after machining, the modules layer the worn tool dataset on top of the reference cutting tool of a similar geometry. The difference gives information related to edge and crater volume removal at the end of tool life. The Mitutoyo SJ-201 portable surface roughness device (Mitutoyo America Corporation, Aurora, IL, USA) was used to measure the workpiece surface roughness an average of 3–4 times as per the ISO 21920-3:2021 standard [37] after every cutting pass.

### 2.4. Characterization and Chip Analysis

The coating thicknesses were measured by placing the samples at an angle in a BC-2 MIBA Coating Group ball crater device (Droitwich Spa, UK). An AISI 52100M Grade 25 mm Chromium steel ball was used to wear away at multiple spots on the coated samples, which were then inspected using a digital microscope. The coatings’ surface topography were obtained using an Anton Parr Tosca^TM^ 400 atomic force microscope (AFM) (Graz, Austria). Line scans were performed over a scan range of 25 µm × 25 µm at a scan speed of 0.3 lines/second using commercial silicon probes. The resonant frequency was set to 278 kHz, and force of 42 N/min. Data were analyzed and processed in Tosca^TM^ analysis software (Version 7.4.8341).

Scratch tests were performed using an Anton Paar-RST3 Revetest^®^ Scratch Tester (Graz, Austria). An average of 3 scratch tests were performed using a 200 µm Rockwell diamond indenter using a 3-scan progressive mode to understand the coatings’ delamination behavior. Pre-topography scan, progressive load scratch scan, and post-topography scan are the three modes that make up the 3-scan procedure. The pre-topography scan gauged the surface profile by applying an initial load of 0.5 N; thereafter, the load was ramped up to 100 N over a distance of 3 mm, with load rate set to ~200 N/min and scan speed to 6 mm/min under progressive load scratch scan, and finally ending off with the post-topography scan for residual depth measurement to extract more details related to the deformation behavior. The morphology and elemental distribution of the scratch tracks were observed with secondary (SE) and backscattered (BSE) modes in a FESEM (FEI, Hillsboro, Oregon, USA and Tescan, Lraya3, Brno, Czech Republic) equipped with an energy-dispersive X-ray spectrometer (EDX).

The micromechanical properties of the mono/bi-layered coatings were evaluated under ambient conditions using a Micro Materials NanoTest P3 system (Wrexham, UK) employing the Oliver–Pharr method. A Berkovich diamond indenter was employed to conduct a minimum of 20 indentations per coating. The indenter was calibrated in accordance with the ISO14577-4 [38] standard, encompassing load displacement, indenter geometry, as well as frame compliance. The indentation contact depth was fixed to 1/10th of the coatings’ thickness such that the substrate effect would be negligible and thus obtain values truly representative of the thin coatings. In other words, the coatings’ load-invariant hardness and elastic modulus were solely considered. A maximum load of 100 mN was achieved at a loading rate of 200 mN/min during the indentation process. This was followed by a dwell time of 2.0 s, and 60.0 s of post-indentation to account for thermal drift.

Three-dimensional surface roughness measurement of the coatings was conducted using the focus variation technology of an Alicona Infinite Focus G5 3D surface measurement system (Alicona Manufacturing Inc., Bartlett, IL, USA). For all the coated samples, an area of ~180,000 µm^2^ was captured at a magnification of 100× with highlights to the peaks and valleys via real color information.

The chemical composition and tribo-oxide formation during the machining process on coated tools’ rake faces were extracted by means of the X-ray photoelectron spectroscopy (XPS) technique using a PHI Quantera II Scanning XPS Microprobe (Physical Electronics Inc., Chanhassen, MN, USA). The spectrometer is equipped with an Al anode source acting as a receiver to electrons targeted towards it via a Raster Scanned Electron Gun^®^, with the generated X-rays redirected towards the samples using a quartz crystal monochromator. Thereafter, the secondary electrons generated pass through a hemispherical energy analyzer in a multi-channel detector to produce secondary electron images (SXI). A monochromatic Al K-α X-ray source in a 50 µm X-ray beam was used to collect data at a constant acceleration voltage of ~1486 eV while being operated at 12.5 W and 15 kV. The max sputtering pressure was reported as 1 × 10^−9^ torr, whereas the lowest base pressure was recorded at 5 × 10^−9^ torr. The spectral data were collected after having sputter-cleaned the samples with Ar+ gas. Spectral survey data were acquired at pass energy of 224 eV in step sizes of 0.8 eV, whereas the high-resolution data were acquired at 55 eV, in step sizes of 0.1 eV. The take-off angle was set to 45° and the dual-beam charge compensation system was utilized to neutralize the coatings. The C1s C-C peak was set to 248.8 eV to calibrate the equipment for high-resolution data acquisition. Post-acquisition data analysis was conducted in PHI Multipak software (Version 9.5).

Chips formed during the machining process were collected after the first pass for each of the coatings to assess their tribological characteristics in relation to the machining process and workpiece surface quality. The chip undersurface roughness, shear band morphology, curliness, and length were observed under a TESCAN VEGA-II LSU SEM. The back surface roughness (S_a_) values for the chips were extracted via an Alicona Infinite Focus G5 3D surface measurement system for all the coated tools and cutting speeds.

## 3. Results and Discussion

### 3.1. Tool Performance and Cutting Forces

**Figure 2:**
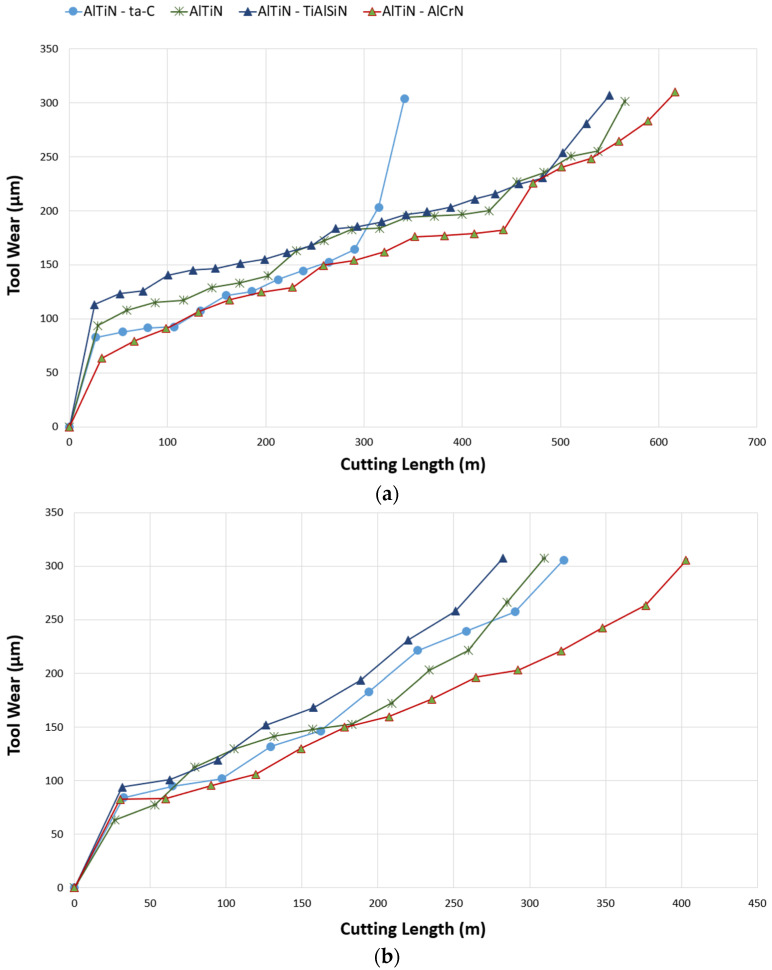

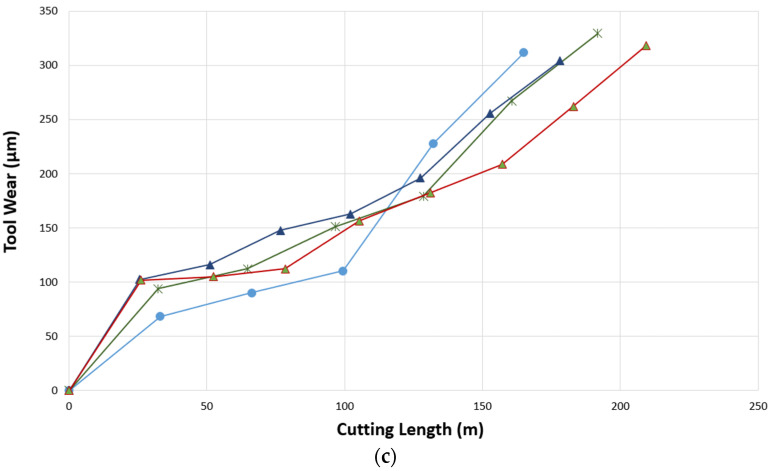
Comparison of tool life curves in terms of flank wear (μm) as a function of cutting length (m) for AlTiN/AlCrN, base AlTiN, AlTiN/ta-C, and AlTiN/TiAlSiN coated tools at the cutting speeds of (**a**) 150 m/min, (**b**) 175 m/min, and (**c**) 200 m/min.

High-speed cutting tests were performed under wet conditions at 150, 175, and 200 m/min to obtain tool life and other complementing data—cutting forces and wear—for the four different coating types. Figure 2 depicts the tool life trends in terms of flank wear as a function of cutting length, with failure classified as flank wear reaching 300 µm. The overall trends across all three speeds supported the AlTiN/AlCrN coating providing the longest tool life, followed by the base layer AlTiN coating, then AlTiN/TiAlSiN, and AlTiN/ta-C being prone to abrupt failure due to its brittle nature. AlTiN/AlCrN had an average tool life improvement of ~19% as compared to the reference monolayer coating when taking all three cutting speeds into account. This improvement in tool life is attributed to its favorable thermomechanical properties (lowest H/E and H^3^/E^2^ ratios, and highest plasticity index (PI)) and tribo-oxide formation as evident from the micromechanical (Table 5) and XPS analysis [39]. The AlTiN/TiAlSiN coating’s tool life was marginally lower across all the speeds when compared to the reference coating. This is owed to the Si inclusion exacerbating the wear rate due to its brittle nature [40], and hence resulting in a shorter tool life. The AlTiN/ta-C coating’s behavior was a lot more unpredictable as it experienced occasional catastrophic failure as a result of its diamond-like nature, with the highest H/E and H^3^/E^2^ ratios, and lowest PI. Overall, as can be observed from Figure 2, for all the coatings, the tool life decremented by approximately 200 m at each instance of increase in speed, which was to be expected, as higher speeds expedite the wear process due to an increase in temperature and stresses. This is evinced by Kaynak Y et al. [26] reporting extreme forces and wear when machining Ti-5553 using carbide tools at cutting speeds above 150 m/min.

**Figure 3:**
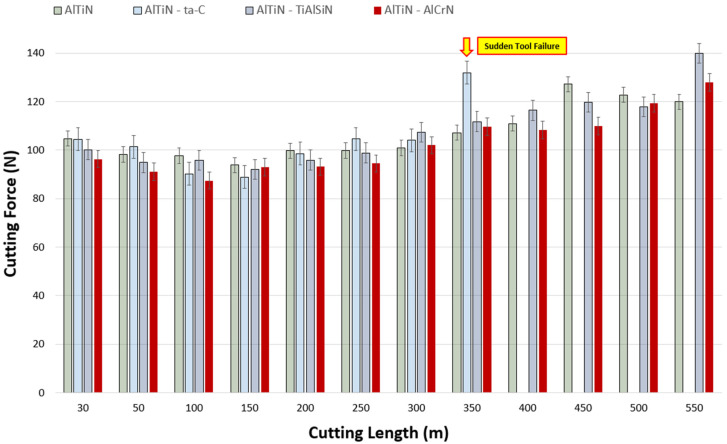
Comparison of cutting force data for AlTiN, AlTiN/ta-C, AlTiN/TiAlSiN, and AlT iN/AlCrN coated tools during the machining of Ti-5553 at the cutting speed of 150 m/min.

**Figure 4:**
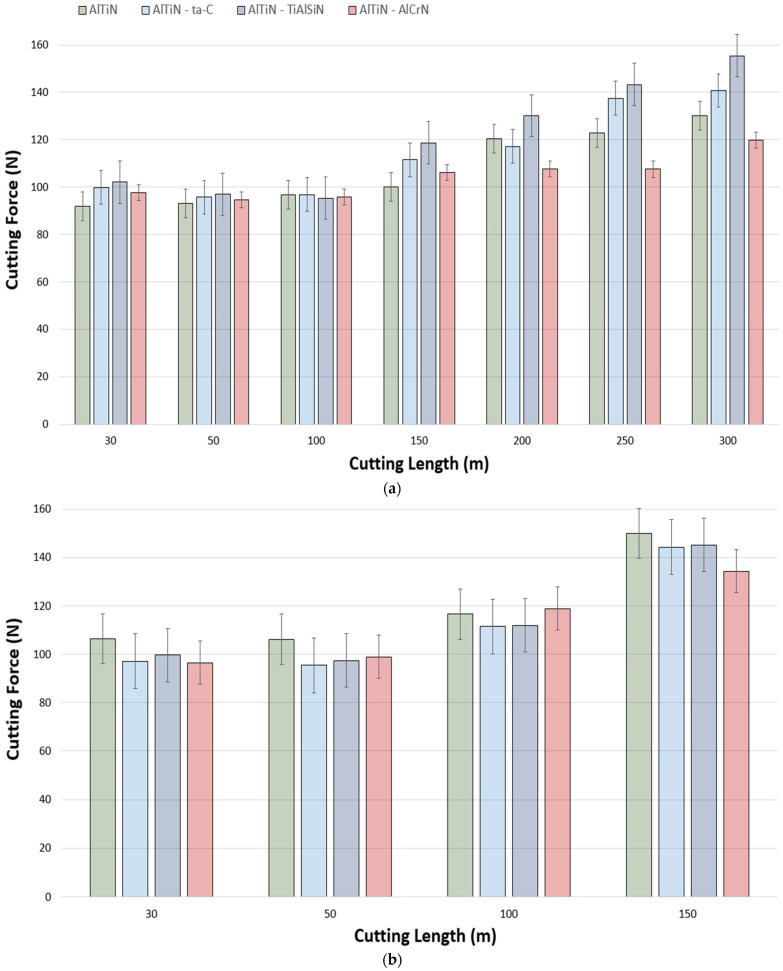
Comparison of cutting force data for AlTiN-, AlTiN/ta-C-, AlTiN/TiAlSiN-, and AlTiN/AlCrN-coated tools during the machining of Ti-5553 at cutting speeds of (**a**) 175 m/min and (**b**) 200 m/min.

Cutting forces typically have a significant impact on the rate of wear progression of a cutting tool, as an increase or decrease in cutting forces is commensurate to changes in frictional forces. Therefore, the higher the cutting forces during the machining process, the worse the frictional characteristics [41]. Cutting forces being high during the machining of superalloys such as Ti-5553 is partially due to their work-hardening nature. Lowering the work-hardening behavior of the workpiece would in turn result in a decrease in cutting forces, temperature, and chatter, and consequently obtaining improved workpiece surface quality and longer tool life. Figure 3 is representative of tangential cutting forces as a function of cutting length at the speed of 150 m/min. It can be noted that, albeit marginal, the lowest cutting forces up to a cutting length of 250 m were obtained by the AlTiN/AlCrN coating. This behavior can primarily be owed to its mechanical properties allowing for higher amounts of plastic deformation before coating delamination and failure. The same trend, however, was not reflected past a cutting length of 250 m, where the behavior was much more unpredictable. The trend for all the coatings was that of cutting forces being an average of 100 N before 250 m of cutting length, and then incrementally increasing by approximately 5 N after each 50 m of cutting length increase until 550 m, where the average force value was found to be 129 N. The rise and unpredictability of cutting forces past the midway point is a result of geometrical distortions at the cutting edge superseding the coatings’ influence on the cutting forces during the cutting process. As indicated by the yellow arrow, a peak of 132 N was observed for AlTiN/ta-C at 350 m of cutting length as it experienced sudden failure due to its extremely hard brittle nature. It is evident from Figure 3 and Figure 4 that across all the speeds, the general trend is that of cutting forces being in the same value range up to the approximate halfway point, and then increasing until the end of life. Moreover, the force variation between the coatings became more unpredictable at the speeds of 175 m/min and 200 m/min due to excessive geometrical deviations at the tool cutting edge. This is further substantiated by the comparatively higher end of life forces found at 175 and 200 m/min: an average of 143 N and 136 N at 200 m/min and 175 m/min, respectively, and 129 N at 150 m/min.

### 3.2. Tool Wear Analysis and Workpiece Surface Quality

**Figure 5:**
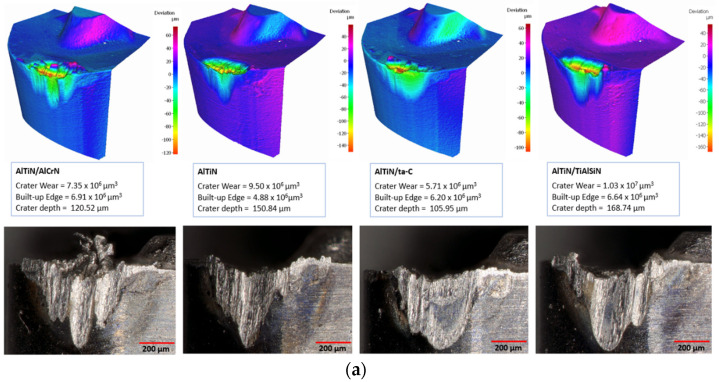

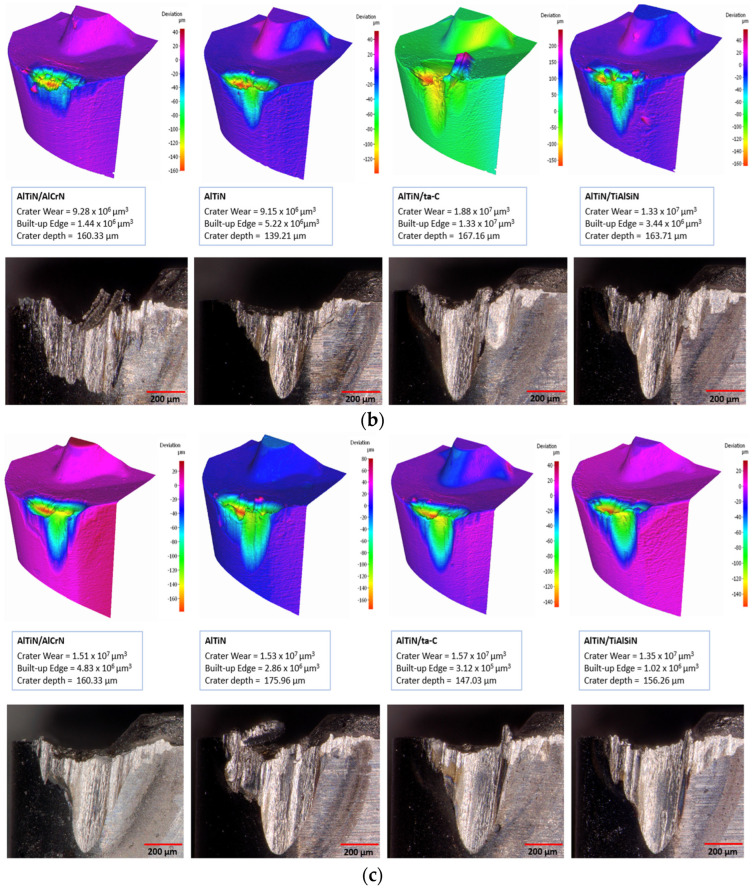
Three-dimensional volumetric datasets and optical images of the coated tools highlighting the amount of crater wear and BUE formation after tool failure (end−of−life) at (**a**) 150 m/min, (**b**) 175 m/min, and (**c**) 200 m/min.

Figure 5 illustrates the end of life volumetrically generated models, and optically taken images of the coated tools at the end of tool life as per the ISO 3685:1993 standard [36]. The two most common wear mechanisms found on all the coatings were flank and crater wear. This was primarily a result of abrasion and attrition forming intense grooves on the cutting edge, allowing for workpiece material to adhere on, and then subsequently being peeled off. Over the many repetitive machining cycles, the adherence and removal of workpiece material from the tools resulted in coating and tool substrate delamination from the flank and rake sides, with each corresponding to flank and crater wear, respectively. The crater wear formation on the rake face is partly a consequence of high temperature generation enabling tool softening and chemical reaction between the tool and workpiece. This leads to diffusion-dominated wear, resulting in crater formation on the rake face of the tool [42,43]. At the speed of 150 m/min, disregarding the abruptly failed AlTiN/ta-C coating, the smallest crater wear and depth were of the AlTiN/AlCrN coating at 7.35 × 10^6^ µm^3^ and 120.52 µm, and largest and deepest crater wear and depth were of the AlTiN/TiAlSiN coating at 1.03 × 10^7^ µm^3^ and 168.74 µm. The AlTiN/AlCrN coating had an advantage in terms of crater wear resistance because it has a relatively high resistance to plastic deformation, stemming from it having the highest PI and lowest H/E ratios. As for AlTiN/ta-C experiencing sudden failure, and AlTiN/TiAlSiN obtaining large and deep crater volumes, this can be attributed to their constituent elements (Si in the case of AlTiN/TiAlSiN, and hard C in AlTiN/ta-C), high H^3^/E^2^ ratios, lesser plastic deformation, and hence proneness to brittle fracture. At higher speeds, however, the end-of-life wear patterns became less predictable and saw a transition from regular flank wear to large and deep notch wears, along with high volume craters, from an average magnitude of ×10^6^ µm^3^ at 150 m/min, to ×10^7^ µm^3^ at 200 m/min. This was to be expected as higher cutting forces and friction during machining cause extreme tool chipping, and hence, as a result, intensified wear rate.

**Figure 6:**
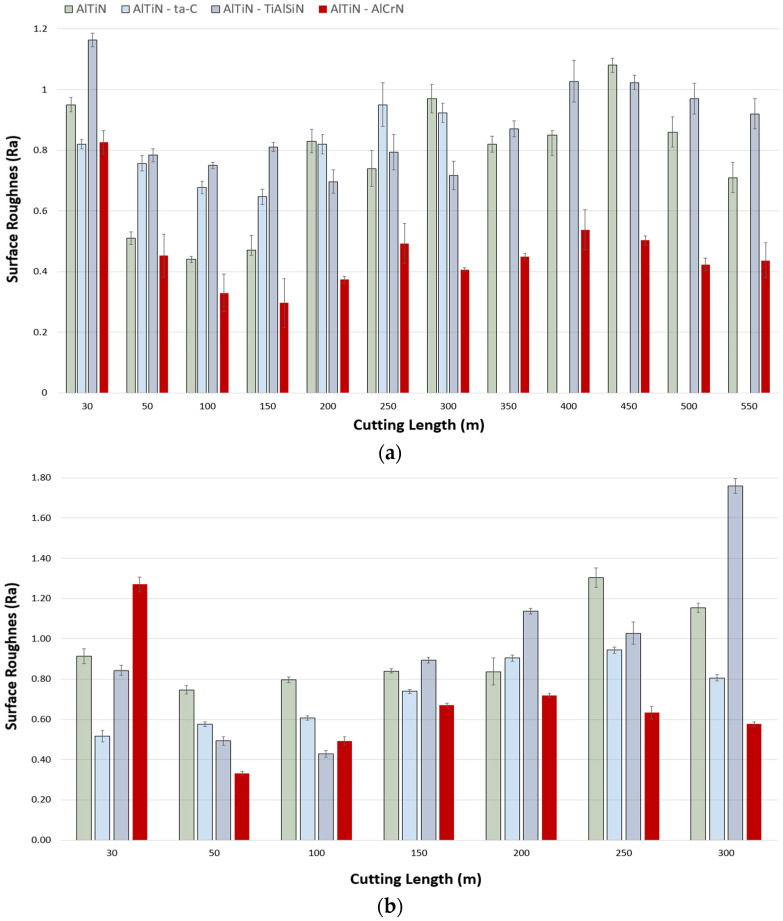

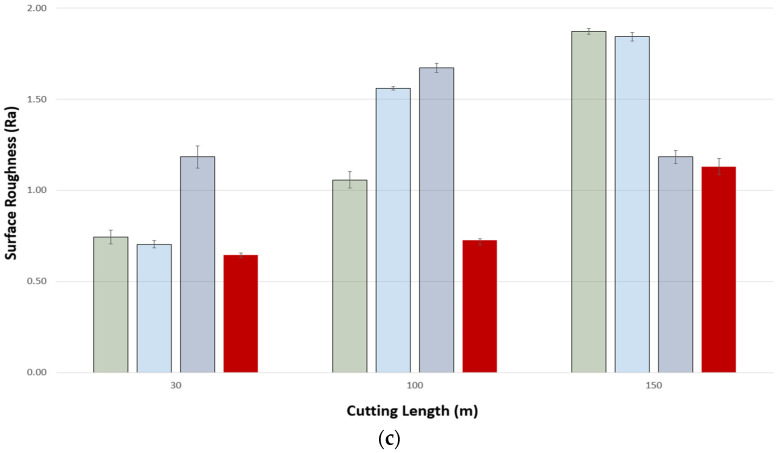
Comparison of surface roughness of the workpiece after machining using the AlTiN/AlCrN-, base AlTiN-, AlTiN/ta-C-, and AlTiN/TiAlSiN-coated tools at the cutting speeds of (**a**) 150 m/min, (**b**) 175 m/min, and (**c**) 200 m/min.

Surface roughness (R_a_) measurements were taken an average of three times at all the cutting speeds after every cutting length pass to get an indication of the workpiece surface quality. Figure 6 depicts the surface roughness measurements taken at the cutting speeds of 150 m/min, 175 m/min, and 200 m/min. Measurements at 150 m/min were taken until close to the end of life (550 m). Overall, it can be noted that there is a decreasing trend of surface roughness values for all the coatings up to a cutting distance of approximately 150 m, with worsened surface quality and unpredictability observed past that distance. This phenomenon is similar to what was observed with the cutting forces, where cutting forces saw a rise and fluctuation past the approximate midway point. The AlTiN/AlCrN immediately stands out as consistently having the lowest surface roughness values throughout the whole cutting process, excluding the first pass (break-in period), decreasing from ~0.83 µm to the lowest value recorded at ~0.30 µm, and then observing an unpredictable trend in the range of ~0.37 µm to ~0.49 µm. It is well established in the literature that AlCrN coatings provide lubricious properties at high temperatures due to the formation of conducive oxides, which aid with reducing friction [44], and in turn result in improved workpiece surface quality. In contrast, the AlTiN/TiAlSiN coating on average imparted the worst workpiece surface quality, ranging from ~0.75 µm to ~1.16 µm, with rising deterioration as cutting length increased. The AlTiN/ta-C coating ranged from ~0.64 µm to ~0.95 µm, with a dipping trend up to 150 m of cutting length, followed by an increase up to its point of sudden failure (300 m). The base reference AlTiN coating too experienced a decreasing surface roughness trend followed by a mean rise and irregularity. A similar general trend of spike after first pass, drop, followed by a rise with irregularity was noted at the speed of 175 m/min as well. The AlTiN/AlCrN coating once again was observed to impart the best surface quality on to the workpiece, with a sharp rise after the first pass (1.27 µm), decrementing to ~0.33 µm, and then increasing and pulsing within the range of ~0.49 µm and ~0.72 µm. The typical trend of the coatings experiencing a break-in period is not apparent from the results at 200 m/min as the high impact of the tool on workpiece negated that effect. Nevertheless, excluding the anomalous behavior of the AlTiN/TiAlSiN coating, there are two important trends to note: all coatings increasing in surface roughness (R_a_), and the AlTiN/AlCrN coating persistently providing the best surface quality.

### 3.3. Micromechanical Properties of the Coatings

This section covers the micromechanical properties of the coatings, as they give a good indication of how a coating would perform during a machining process, and serve as a basis to substantiate and explain the observed results. Table 5 provides details of the coatings’ micromechanical properties, architecture, thickness, surface roughness, and other relevant properties. The coatings’ H/E and H^3^/E^2^ ratios were calculated from the hardness (H) and elastic modulus (E) values obtained via a nanoindenter. These properties are often utilized to make inferences about a coating’s resistance to wear. The H/E ratio represents a materials elastic strain to failure, or the measure of elasticity at the point of contact between the indenter tip and coating in this context, whereas the H^3^/E^2^ ratio is the coating’s ability to resist plastic deformation. It has been reported that under heavy load cutting applications, with the dominant wear mechanism being adhesion, a stable adhesive layer forms on coatings with low H/E and H^3^/E^2^ ratios, resulting in reduced frictional forces [45]. This trend is the opposite of what is typically found—the higher the H/E and H^3^/E^2^ ratios, the better the wear resistance. Another indicator to consider is the plasticity index (PI), which is calculated based on the proportion of plastic work to the total work during indentation [46]. A higher PI indicates that the coating experienced more plastic deformation during indentation. In scenarios where abrasion is dominant, a high H/E ratio (indicating a lower PI) is usually desirable. However, in operations where adhesive wear is dominant, the opposite trend is observed. Studies [47] suggest that having a reserve of plasticity is necessary to dissipate the energy produced during friction, with a higher PI correlating with greater energy dissipation. Additionally, the findings of [48,49] support this trend in turning and milling operations, respectively, where adhesive wear is dominant. Hence, the AlTiN/AlCrN coating having the smallest of these H/E and H^3^/E^2^ ratios and the highest PI translates to it attaining the best tool-life and workpiece surface quality. The AlTiN/ta-C coating, on the other hand, has the opposite trend, and that translates to it experiencing brittle failure during the machining process, and chips with staggered ridges and roughness within the spacings. The AlTiN/TiAlSiN coating had marginally higher hardness than the base AlTiN coating and had similar H/E and H^3^/E^2^ ratios. These properties when translated to the machining process entails the AlTiN/TiAlSiN coating having marginally shorter tool life as compared to the base monolayer coated tool. Other characterization tests discussed in the Materials and Methods Section have been discussed elsewhere by Syed et al. [39].

### 3.4. Tribological Performance

**Figure 7:**
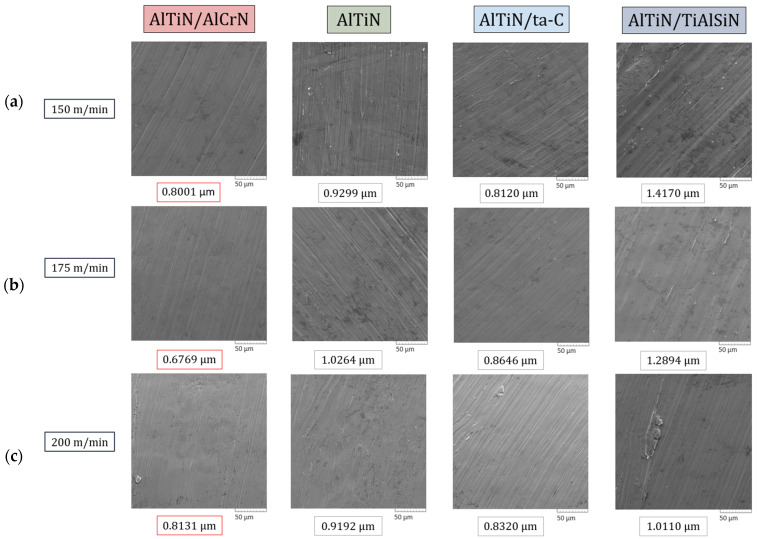
SEM images and surface area roughness (Sa) values of chips collected after the first pass during the machining of Ti-5553 with AlTiN/AlCrN-, base AlTiN, AlTiN/ta-C-, and AlTiN/TiAlSiN-coated tools at the cutting speeds of (**a**) 150 m/min, (**b**) 175 m/min, and (**c**) 200 m/min. Magnification 1000×.

Chip tribological analysis is essential to perform machining at high cutting speeds as it adds an additional layer of depth to the understanding of tool–workpiece interaction and the associated variables—tool life, friction, and the workpiece surface quality imparted as a result [50]. An SEM was used to take high magnification images of the chips to characterize the back surface roughness, shear band morphology, and shape as they tend to be an excellent guide to gain insight into the tribological behavior at the tool–chip interaction zone. Figure 7 shows the visual representation of the back surface quality (magnification 1000×) and accompanying surface roughness (S_a_) values extracted via an Alicona Infinite Focus G5 3D surface measurement system for all the coated tools and cutting speeds. By a visual inspection of the SEM images, several key features can be noted at each of the three cutting speeds: The AlTiN/AlCrN coating being a softer coating, with its micromechanical properties conducive to allowing for prolonged plastic deformation, formed chips that had the smoothest back surface roughness and minimal crease lines. At the highest speed of 200 m/min, due to thermal deformation and stresses, small pockets of uneven spots started to appear. Chips collected from the initial pass of the AlTiN coating indicate that it experienced extensive tearing and had uneven spots as a result of chips sticking to the tool’s surface [50,51]. The chips collected from machining via the AlTiN/ta-C coated tool had abrasion and occasional appearance of shred marks as the primary damage mechanism. This is owed to the AlTiN/ta-C coating having a relatively higher coefficient of friction as indicated in a previous work [39]. Chips formed via machining of Ti-5553 using AlTiN/TiAlSiN coated tools had shred marks and folds as the main mechanism of deterioration.

Visual inspection is only one of the many methods by which chip analysis can be performed. Back surface roughness (S_a_) is another key indicator to help further understand the tribological behavior at the tool–chip interaction zone during a turning machining process. The general pattern is that of the back surface roughness values being in a particular range for each of the respective coatings: the worst chip back surface quality was found to be on the chips produced using the AlTiN/TiAlSiN coating, with an average value of ~1.239 μm, followed by the base AlTiN coating at ~0.959 μm, AlTiN/ta-C coating at ~0.836 μm, and AlTiN/AlCrN coating having the smoothest back surface roughness at ~0.763 μm. In a recent study by the authors [39], XPS analysis was performed on the rake face of the tools to understand diffusion/oxidation interaction between the workpiece and the cutting tool. The low surface roughness of the chips obtained by the AlTiN/AlCrN coating can partly be attributed to the formation of beneficial Cr_2_O_3_ and (Al, Cr)_2_O_3_ (due to Cr_2_O_3_ solubility in alumina) tribo-oxides. These tribo-oxides have been reported to provide lubricious and thermal barrier properties at high temperatures, which help with reducing friction at the tool–chip interaction zone and protect the tool substrate from thermal damage.

**Figure 8:**
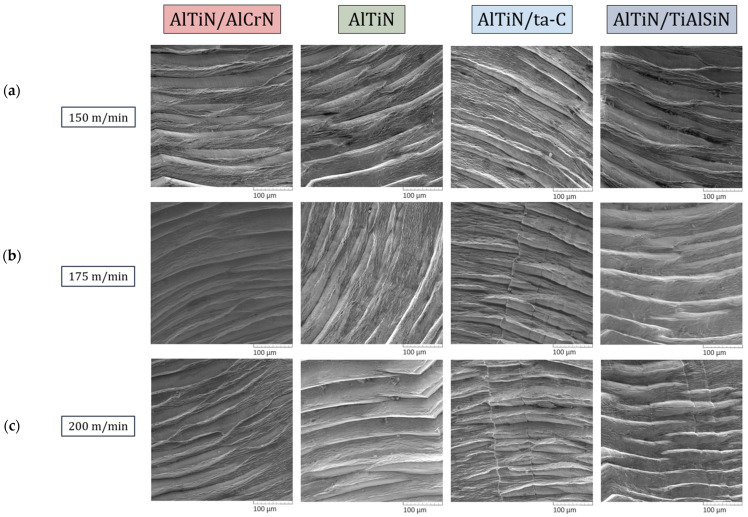
Shear band morphological comparison of chips collected after the first pass during the machining of Ti-5553 with the AlTiN/AlCrN-, base AlTiN-, AlTiN/ta-C-, and AlTiN/TiAlSiN-coated tools at cutting speeds of (**a**) 150 m/min, (**b**) 175 m/min, and (**c**) 200 m/min. Magnification 600×.

Shear bands of the chips shown in Figure 8 are of significance as they reveal information pertaining to the flow of chips as well as deformation behavior. The shear bands produced by the AlTiN/AlCrN coating experienced minor splitting indicative of plastic deformation with spacing between the ridges holistically smoother than the other three coatings. This relatively better morphology in the AlTiN/AlCrN chips may be owed to it having undergone prolonged plastic deformation and smoother chip flow due to low friction at the tool¬–chip interface. The chips produced by the base AlTiN-coated tools had a tendency to experience extreme anisotropic deformation with inconsistent spacing between the ridge segments. In other words, the chip flow and deformation were not continuous. Shear bands collected after machining via the AlTiN/ta-C-coated tools had staggered segments with a continuous ridge line across the shear bands, and rough spacing in-between. The AlTiN/TiAlSiN coated tools produced chips that had an unpredictable pattern across the speeds: large spacing between the ridges at 150 m/min, variance in ridge sizes at 175 m/min, and staggered behavior at 200 m/min.

## 4. Conclusions

This study investigated the high-speed finish-turning—150 m/min, 175 m/min, and 200 m/min—of Ti-5553 using novel mono/bi-layered WC cutting tools to fill a void that exists in the literature. The cutting tools involved are composed of a base monolayered AlTiN-coated tool, and AlCrN, ta-C, and TiAlSiN coatings each deposited on top of the base AlTiN coating on three separate cutting tools to form bi-layered AlTiN/AlCrN, AlTiN/ta-C, and AlTiN/TiAlSiN coatings, respectively. The following conclusions are drawn based on the analysis:

- A comparative analysis indicated that the AlTiN/AlCrN-coated tools obtained the longest tool life with an average improvement over the base reference of about 19% based on the average of the three cutting speeds.
- The AlTiN/ta-C coating’s brittle nature led to it experiencing premature failure.
- The general trend for all the coatings was that of cutting forces remaining within a specified range up to the approximate midway point of tool life, after which a rise was observed due to geometrical deviations at the cutting tool edge.
- The mean lowest cutting force values obtained by the AlTiN/AlCrN-coated tools can be attributed to its optimal micromechanical properties permitting prolonged plastic deformation before failure.
- The most common wear mechanisms across the speeds were flank and crater wear, with the AlTiN/AlCrN coated tool having experienced the smallest crater wear volume and depth at 150 m/min.
- In general, all the coated tools experienced progressively worsening crater and flank wear with increasing speed, particularly at 200 m/min.
- The AlTiN/AlCrN-coated tools consistently, at all speeds, imparted the highest surface finish on the workpiece, whereas the AlTiN/TiAlSiN-coated tools frequently imparted the worst.
- The chip back surface roughness values were found to be within a specified range for each of the respective coatings; similar to what was observed with the workpiece surface roughness, the smoothest back surface roughness was that of chips obtained via machining using the AlTiN/AlCrN-coated tool as a result of conducive oxide formation and optimal micromechanical properties, and the worst by the AlTiN/TiAlSiN-coated tool due to its hard nature and constituent elements.

## Figures and Tables

**Table 1:** Chemical composition of Ti-5553 and Ti-64 at ambient temperature [7,10,13,14].

Composition (wt.%)	Al	Mo	V	Cr	Fe	Z	O	N	C
Ti-5553 alloy	4.5–6	4–5.5	4–5.5	2.5–3.5	0.3–0.5	<0.4	<0.2	<0.1	<0.1
Ti-64 alloy	4.5–6	-	3.5–4.5	-	0.3–0.8	-	<0.2	<0.1	<0.1

**Table 2:** Thermomechanical properties of Ti-5553 and Ti-64 at ambient temperature [7,10,13,14,15,16,17].

Properties	Tensile Strength (MPa)	Yield Strength (MPa)	Elongation (%)	Hardness (HV)	Thermal Conductivity (W/m^−1^C^−1^)
Ti-5553 alloy	1290	1170	6	415	5
Ti-64 alloy	~950	~880	14–18	~300	6.7–7.3

**Table 3:** Summary of existing research on turning machining of Ti-5553.

Paper Details			Cutting Tool and Workpiece		Machining Conditions		Testing Outputs		
No.	Reference	Objective	Cutting Tool Material	Coating	Feed (f), Depth of Cut (a_p_), Speed (V_c_)	Coolant	Thermomechanical	Visual	Important Conclusions
1.	Kaynak Y. [23]	To analyze the impact of cutting parameters on machining of Ti-5553	883 Carbide—CNMG120408 M1	Uncoated	V_c_: 40, 80, 120, 160, 200 m/min a_p_: 0.8, 1.4, 2 f: 0.1, 0.15, 0.2 mm/rev	Dry	Surface roughness, cutting forces	Tool wear, cutting temperature, chips	Depth of cut and cutting speed have a profound effect on the machining performance Feed rate has minimal effect on the machining performance, but does impact the surface roughness of the material Common wear mechanism is BUE on the rake face Nose wear increases considerably above 120 m/min
2.	Sun Y. [22]	Assessing different cutting fluid mechanisms on machining performance of Ti-5553	SANDVIK 432-MM (Carbide)	* Coated (TiCN)	V_c_: 20, 50, 80 m/min f: 0.05, 0.125, 0.2 mm/rev	Cryo (10 g/s, 1.5 MPa), flood, and MQL	Cutting forces	Tool wear, surface roughness	Cutting forces were reduced by 30% with cryogenic cooling as compared to flood and MQL Nose wear reduced in cryogenic cooling due to reduced adhesion of material The lowest surface roughness values were found by using MQL due to high temperature and lubricity effects resulting in softening of the workpiece Cutting forces increased with feed rate Flank wear was insensitive to cooling conditions and was found in all Adhesion was the primary wear mechanism of the nose
3.	Tascioglu E. [23]	Assessing different cutting fluid mechanisms on machining performance of Ti-5553	883 Carbide—CNMG120408 M1	Uncoated	V_c_: 90 (dry and lubrication), 120 (cryogenic) m/min a_p_: 0.6 (finishing), 1.2 (roughing) f: 0.15 mm/rev	Dry, flood (400 L/h), HPC (50 bar), MQL (21 mL/h, 0.4 MPa), and cryo (10 g/s, 15 bar)	Cutting forces	Tool wear, cutting temperature, chips, surface roughness	Greatest tool life observed under HPC, followed by cryo HPC was found to be the best coolant in reducing friction and temperature, and consequently wear, and cutting forces Cryo cooling is more effective at lower depths of cut (finishing)
4.	Kaynak Y. [24]	Assessing different cutting fluid mechanisms on machining performance of Ti-5553	883 Carbide—CNMG120408 M1	Uncoated	V_c_: 30, 90, 120, 150, 210 m/min a_p_: 1.2 f: 0.15 mm/rev	Flood (400 L/h, MQL (21 mL/h, 5 bar), and HPC (50 bar)	Cutting forces, microhardness,	Tool wear, chips, surface roughness	Beyond 150 m/min extreme wear and forces were observed HPC provides the best results Reduced hardness observed after machining No strain hardening observed Phase transformation does not occur hence post-treatment is not needed
5.	Yan D.P. [25]	Examining chip formation, and calculating shear strains, and rates	WNMG carbide	Uncoated	V_c_: 37, 49, 66 m/min a_p_: 0.2 mm f: 0.05, 0.1, 0.2 mm/rev	Dry	Shear stress and strain	Chip formation	Shear strain within the bands was observed to be greater than in segments Shear strain rate increased with cutting speed Shear strain increased with material removal rate (MRR)
6.	Kaynak Y. [26]	Assessing different cutting fluid mechanisms on chip formation of Ti-5553	TCMW16T308H13A (Carbide)	Uncoated	V_c_: 20, 60, 120, and 210 m/min f: 0.1 mm/rev	Cryo (15 bar), MQL (21 mL/h), and HPC (50 bar)	Cutting forces	Cutting temperature, tool wear, chip formation	Increased cutting speeds lead to segmented chips in all conditions Chip thickness was larger at low cutting speeds Cryogenic condition produced the thinnest chips and smallest tool–chip contact length Shear angle becomes larger with increased cutting speeds resulting in thinner chips Cutting forces decreased with increasing cutting speeds Only HPC helped with improving chip breaking
7.	Liu E. [27]	A novel approach to assess tool wear	CNMG120408-SMRH13A (Carbide)	Uncoated	V_c_: 30, 60, 90 m/min a_p_: 0.5, 1, 1.5 mm f: 0.05, 0.1, 0.15, 0.2, 0.25 mm/rev	Cryo (5.5 g/s)		Tool wear (Keyence for wear modes, SEM for microscopic wear morphology, EDS to analyze wear modes)	Primary wear mechanisms on rake face were BUE, BUL, crater, notch, chip flow damage, and burr damage
8. *	Arrazola P.-J. [10]	Machinability comparison between Ti-5553 and Ti-64	CNMG 120408-2 (Carbide)	Uncoated	V_c_: 40-60 (Ti-5-5-5-3), 90 (Ti-6-4) m/min a_p_: 2 mm f: 0.1 mm/rev	Conventional	Cutting forces	Tool wear, chips	Machinability of Ti-64 was observed to be higher than Ti-5553 Higher cutting forces when machining Ti-5553 SEM observations of the worn tool detected the presence of titanium carbide. The presence of carbon in the adhered material on the tool indicates diffusion of carbon from insert to workpiece
9.	Wagner V. [6]	Effect of cutting parameters during machining of Ti-5553	Tungsten carbide	Coated (TiAlN)	V_c_: 35-65 m/min a_p_: 3 mm f: 0.2 mm/rev	Dry	Cutting forces	Tool wear, chips	
10.	Wang L. [28]	Chip morphology comparison between dry and HPC in machining of Ti-5553	CNMG 432-SMR H13A	Uncoated	V_c_: 30, 60, 120 m/min a_p_: 0.5 mm f: 0.1, 0.2 mm/rev	Dry, and HPC (105 bar)		Chips	HPC improved machinability Better chip morphology and chip breaking under HPC Serrated chips (adiabatic shear phenomenon) more severe under HPC The degree of chip sawtooth intensifies with increasing cutting speed and feed rate HPC chips had a mix of ductile + quasi-cleavage fracture whereas dry cutting chips had ductile fracture
11.	Liu E. [29]	A novel approach to assess tool wear	CNMG120408-SMRH13A (Carbide)	Uncoated	V_c_: 30, 60, 90 m/min a_p_: 0.5, 1, 1.5 mm f: 0.05, 0.1, 0.15, 0.2, 0.25 mm/rev	Cryo (5.5 g/s)		Tool wear (Keyence for wear modes, SEM for microscopic wear morphology, average value of flank wear VB to evaluate wear)	
12.	Kaynak Y. [30]	Cryogenic coolant to assess machinability of Ti-5553	883 Carbide—CNMG120408 M1	Uncoated	V_c_: 30, 90, 120, 150, 210 m/min a_p_: 1.2 f: 0.15 mm/rev	Dry, and cryo (Ln2: 1.5 MPa, 10g/s CO2: 5.4 MPa)	Cutting forces, surface integrity	Cutting temperature, tool wear	Up to 120 m/min similar tool wear was observed for all 3 conditions In terms of material removal rate and cutting force 120 m/min was deemed the best Cryogenic cooling shortened chip contact length and improved breakability Reduced dimensional deviation in cryogenic due to reduced wear Surface and subsurface hardness of the workpiece decreased under all conditions XRD showed that the crystal structure and phases on the surface were affected during machining Cutting forces increased above 120 m/min due to tool wear
13.	Yan D.P. [31]	Investigated chip formation mechanisms, morphology, and microstructure evolutions	WNMG carbide	Uncoated	V_c_: 27, 39, 55 m/min a_p_: 2 mm f: 0.08, 0.11, 0.2 mm/rev	Dry	Shear strain, strain rate	Chips, shear angle, shear	Chip serration occurs during high-speed machining of Ti-5553, which is found to be attributed to the periodic formation of shear bands caused by thermoplastic instability within the primary shear zone. Effect of feed rate was found to be greater than cutting speed on shear angle
14.	Zhao X. [32]	Effect of surface integrity on fatigue life under cryogenic cooling in machining of Ti-5553 is analyzed	CNMG 12 04 08-SMR H13A	Uncoated	V_c_: 30, 60, 90 m/min a_p_: 0.5 mm f: 0.10, 0.20 mm/rev	Dry and cryo (0.09 MPa)	Surface roughness, surface topography, fatigue, residuals stress		As the cutting speed increased from 30-90 m/min the surface roughness first decreased followed by an increase At constant cutting speed, surface roughness increased with increasing feed Cryogenic cooling extended fatigue life
15.	Liu E. [33]	Experimental and numerical approach to studying the effect of dry and cryogenic machining of Ti-5553	CNMG 12 04 08-SMR H13A	Uncoated	V_c_: 50, 60 m/min a_p_: 0.2, 0.4, 0.6, 0.8 mm f: 0.10, 0.12, 0.14, 0.16, 0.18, 0.20 mm/rev	Dry and cryo		Cutting temperature	At 50 and 60 m/min, and ap = 0.8 mm, an increase in feed rate under both cryo and dry conditions results in temperature increase (simulation and experimental) Marginal increase in temperature with change in depth of cut
16.	Liu E. [34]	Effect of different cooling mechanisms on the surface integrity during machining of Ti-5553	CNMG1204 08-SMR H13A	Uncoated	V_c_: 30, 60, 90 m/min a_p_: 0.5 mm f: 0.10, 0.15, 0.20 mm/rev	Dry, cryo, (0.09 MPa) and HPC (8 MPa)	Surface roughness, plastic deformation, work hardening, residual stresses		As the cutting speed increased the surface roughness first decreased followed by an increase At constant cutting speed, surface roughness increased with increasing feed Surface hardness is mostly affected by cutting speed, followed by the cooling condition, and finally feed rate Max tensile residuals stress under dry whereas max compressive residual stress under HPC condition
17.	Liu E. [35]	A numerical approach to assess surface morphology of Ti-5553	CNMG120408-MJ	Uncoated	V_c_: 30, 50, 70 m/min a_p_: 0.5 mm f: 0.05, 0.15, 0.25 mm/rev	Dry, and cryo	Cutting forces	Surface roughness, surface morphology	Cryogenic cooling affects low- and high-frequency surface roughness Cutting parameters affect low-frequency surface roughness Better 3D surface morphology found in cryo than in dry Increase in cutting speed led to decrease followed by an increase of low-frequency surface roughness Increase in feed resulted in the increase of low- and high- frequency surface roughness

* Indicates use of both Ti-64 and Ti-5553 workpieces. Green background indicates the use of a coating.

**Table 4:** Cutting parameters and conditions.

Cutting Conditions				
Cutting Speed (m/min)	Feed Rate (mm/rev)	Depth of Cut (mm)	Coolant Type	Workpiece Material
150	0.15	0.25	Castrol Hysol MB50 Semi-Synthetic (9%)	Ti-5553 Grandis Titanium
175				
200				

**Table 5:** Mechanical, architectural, and surface properties of the coated tools.

Coating Type	AlTiN	AlTiN/AlCrN	AlTiN/ta-C (DLC)	AlTiN/TiAlSiN
Architecture	Monolayer	Monolayer	Monolayer	Monolayer
Thickness (µm)	5.57	6.6	6.05	6.54
Hardness (GPa)	33.87 ± 3.78	24.23 ± 4.96	41.88 ± 6.46	36.44 ± 6.73
Elastic modulus (GPa)	503.84 ± 47.25	450.35 ± 72.24	487.54 ± 71.60	588.01 ± 112.56
H/E	0.0672	0.0538	0.0859	0.0620
H^3^/E^2^	0.153	0.070	0.309	0.140
Plasticity index (PI)	0.470	0.515	0.354	0.473
S_a_ (nm)	36.91	33.59	43.47	45.38

## Data Availability

All data are presented in the current manuscript. For further queries, communicate with the corresponding authors.
